# Prototype Features for a Sleep-Enhancing Music App in Older Adults Living with Dementia

**DOI:** 10.1093/geroni/igaf122.3188

**Published:** 2025-12-31

**Authors:** Osborn Owusu Ansah, Justine Sefcik, Nancy Hodgson, Bei Wu, Darina Petrovsky

**Affiliations:** Duke University, Durham, North Carolina, United States; Drexel University College of Nursing and Health Professions, Philadelphia, Pennsylvania, United States; University of Pennsylvania, Philadelphia, Pennsylvania, United States; NYU Shanghai, Shanghai, China (People’s Republic); Duke University, Durham, North Carolina, United States

## Abstract

Music interventions show promise for addressing insomnia in persons living with dementia (PLWD), as musical memory often remains intact despite cognitive decline and can be personalized to individual preferences. The study aimed to identify the prototype features of the mobile application, “Calming Music Personalized for Sleep Enhancement in PeRsons living with Dementia” (CoMPoSER) for PLWD and their caregivers. We conducted two rounds of interviews in a collaborative design process. In round one, we held 13 virtual interviews (N = 20), including 6 PLWD and 14 caregivers (PLWD average age 80 years old, SD = 12.1; caregiver average age 61, SD = 16.1). Seven interviews were conducted with both PLWD and their caregivers, and six were conducted with caregivers only. Most were women (n = 14) and 12 out of 20 (60%) identified themselves as White/Caucasian. In round two, we conducted 7 virtual interviews with 10 participants returning from the first round (2 PLWD and 8 caregivers) using the mHealth Usability framework and directed content analysis. We asked participants to select their preferred features and layout options for the CoMPoSER application. When presented with five visual design options, participants recommended labeled symbols, simple layouts, and tutorial options for those with memory problems. Most preferred darker blue color schemes to promote sleep and limited customization options. These insights directly informed the mobile application design, which will be presented.

